# Copy Number Variation of Cytokinin Oxidase Gene *Tackx4 *Associated with Grain Weight and Chlorophyll Content of Flag Leaf in Common Wheat

**DOI:** 10.1371/journal.pone.0145970

**Published:** 2015-12-29

**Authors:** Cheng Chang, Jie Lu, Hai-Ping Zhang, Chuan-Xi Ma, Genlou Sun

**Affiliations:** 1 College of Agronomy, Anhui Agricultural University; Key Laboratory of Wheat Biology and Genetic Improvement on Southern Yellow & Huai River Valley, the Ministry of Agriculture, Hefei, 230036, China; 2 Department of Biology, Saint Mary’s University, Halifax, NS B3H3C3, Canada; Murdoch University, AUSTRALIA

## Abstract

As the main pigment in photosynthesis, chlorophyll significantly affects grain filling and grain weight of crop. Cytokinin (CTK) can effectively increase chlorophyll content and chloroplast stability, but it is irreversibly inactivated by cytokinin oxidase (CKX). In this study, therefore, twenty-four pairs of primers were designed to identify variations of wheat CKX (*Tackx*) genes associated with flag leaf chlorophyll content after anthesis, as well as grain weight in 169 recombinant inbred lines (RIL) derived from *Triticum aestivum* Jing 411 × Hongmangchun 21. Results indicated variation of *Tackx4*, identified by primer pair T19-20, was proven to significantly associate with chlorophyll content and grain weight in the RIL population. Here, two *Tackx4* patterns were identified: one with two co-segregated fragments (*Tackx4-1*/*Tackx4-2*) containing 618 bp and 620 bp in size (as in Jing 411), and another with no PCR product. The two genotypes were designated as genotype-A and genotype-B, respectively. Grain weight and leaf chlorophyll content at 5~15 days after anthesis (DAA) were significantly higher in genotype-A lines than those in genotype-B lines. Mapping analysis indicated *Tackx4* was closely linked to *Xwmc169* on chromosome 3AL, as well as co-segregated with a major quantitative trait locus (QTL) for both grain weight and chlorophyll content of flag leaf at 5~15 DAA. This QTL explained 8.9~22.3% phenotypic variations of the two traits across four cropping seasons. Among 102 wheat varieties, a third genotype of *Tackx4* was found and designated as genotype-C, also having two co-segregated fragments, *Tackx4-2* and *Tackx4-3* (615bp). The sequences of three fragments, *Tackx4-1*, *Tackx4-2*, and *Tackx4-3*, showed high identity (>98%). Therefore, these fragments could be considered as different copies at *Tackx4* locus on chromosome 3AL. The effect of copy number variation (CNV) of *Tackx4* was further validated. In general, genotype-A contains both significantly higher grain weight and flag leaf chlorophyll content at 5~15 DAA than those in genotype-B and genotype-C, among 102 varieties under various environments.

## Introduction

Photosynthesis, which provides raw material for plant products, is pivotal to food and fiber production [1]. Under favorable conditions, approximately 70–90% final grain yield is derived from photosynthates produced during the grain-filling period [2, 3]. In high plant leaves, chlorophyll, including chlorophyll *a* and *b*, is the main photosynthetic pigment in chloroplasts, and its amount directly affects plant photosynthetic efficiency [4–5]. Increased chlorophyll content in crop-species leaves increases in both biomass production and grain yield [6]. As chlorophyll is the main pigment in photosynthesis, its abundance and stability in the leaf significantly affects grain filling and crops yield [6–10]. Therefore, understanding both chlorophyll metabolism and genetic mechanism controlling chlorophyll content is important for improved crops yield. Research suggested that cytokinin (CTK), a phytohormone, can greatly increase leaf chlorophyll content, chloroplast stability, and net photosynthetic rate [11–21]. Transgenic plants overexpressing the isopentenyl transferase gene (*ipt*) have generally shown a stay-green phenotype because of a high level of CTK synthesized in vivo [22, 23]. As for the regulation of CTK level in plants, CKX plays a key role in inactivating CTK levels irreversibly by cleaving the N^6^-side chain. Previous research confirmed that CKX is involved in chlorophyll level and photosynthesis regulation by controlling plant CTK content [24, 25]. Therefore, *CKX* gene can be presumed to have a close relationship with chlorophyll level and stability.

Most studies generally focus on associations between variations of *CKX* genes and grain yield and related traits in cereal [26–29]. In wheat, seven *Tackx* genes have been isolated, including *Tackx1* on chromosome 3A [30]; *Tackx2* on 7A or 7B [31]; *Tackx2*.*1* and *Tackx2*.*2* on 3DS [28]; and *Tackx3* [32]; *Tackx5* [33]; and *Tackx6* [29] on 3DS. These *Tackx* genes are usually associated with grain weight [28] or grain numbers per wheat spike [29]. However, little is known about the association of *Tackx* gene with both wheat chlorophyll content and grain weight. The objectives of this study were to: (1) identify variations in *Tackx* and their association with grain weight and wheat chlorophyll level, and (2) validate the effect of target *Tackx* gene on these two traits.

## Materials and Methods

### Plant Materials

One hundred and sixty-nine recombinant inbred lines (RIL) were obtained from a cross between Jing 411 and Hongmangchun 21 using single-seed descent (F_2:8_ generation) method. Jing 411 is a winter-type variety with deep-green flag leaf, high yield, and large grains, with an averaged thousand-grain weight (TGW) of 46.2 g, based on data from four cropping seasons (2009–2010, 2010–2011, 2011–2012, and 2012–2013). Hongmangchun 21, a Chinese landrace, is a spring-type variety with low TGW (20.9 g) (Table 1). The two parents showed significant difference in both grain weight and flag leaf chlorophyll content (Table 1). To further examine the effect of *Tackx* on grain weight and flag leaf chlorophyll content, 102 wheat cultivars with significant difference in grain weight and flag leaf chlorophyll content were also analyzed.

**Table 1:** Chlorophyll content and grain weight of the two parents and RIL population based on averaged values from four cropping seasons.

Trait^a^	Parents		RIL population (*n* = 169)		Natural population (*n* = 102)	
	Jing411	Hongmangchun 21	Mean± SD	C.V.%	Mean± SD	C.V.%
C5	50.66	36.08	48.92±4.58	9.36	50.27±3.62	7.20
C10	50.44	32.13	48.49±4.90	10.11	47.09±3.51	7.46
C15	50.02	28.14	44.61±7.99	17.93	42.35±5.54	13.09
C20	42.39	16.35	29.49±11.5	38.99	28.74±11.84	41.18
C25	1.38	9.80	7.21±4.33	60.12	0.74±0.31	42.03
TGW	46.2	20.9	36.15±7.65	21.16	36.72±4.81	13.11

a: C5, C10, C15, C20, and C25 represent flag leaf chlorophyll content (SPAD value) at 5, 10, 15, 20, 25 days after anthesis, respectively. TGW: thousand-grain weight.

### Field Trials

Of the 102 wheat varieties, RIL and their parents were grown in randomized, complete blocks with two replicates at the Experimental Station of Anhui Agricultural University (Hefei, 31°58′N, 117°24′E) in cropping seasons 2009–2010, 2010–2011, 2011–2012, and 2012–2013. Each plot contained three, 2.0 m rows spaced 25 cm apart, with 40 plants in each row. Common field management practices for wheat production were followed. Average rainfall, temperature, and sunlight were 950 mm, 15.5°C and 2,100 h per year across the four seasons, respectively.

### Measurements of Grain Weight and Relative Chlorophyll Content in Flag Leaf

TGW was measured by weighing two samples of 1000 kernels for each line. Chlorophyll content of the RILs and 102 wheat varieties across the four cropping seasons were measured using methods reported in the previous studies [34, 35] with minor modification. Chlorophyll content was measured as the SPAD value using a chlorophyll meter (SPAD 502, Minolta, Osaka, Japan). For each wheat lines, flag leaves from 10 randomly-chosen main tillers (fully extended leaf without disease) were used for SPAD measurements. Five SPAD readings, randomly sampled from flag leaf tip to base, were averaged to obtain a value for each individual plant. Chlorophyll content was measured at 5, 10, 15, 20, and 25 days after anthesis (DAA), and chlorophyll contents at the five stages were designated as C5, C10, C15, C20, and C25, respectively.

### Genomic DNA Extraction and Polymerase Chain Reaction Amplifications

Genomic DNA was extracted from two kernels per line according to the previous publication [36]. Polymerase chain reactions (PCR) were conducted on a TC412 Thermocycler (Barloworld Scientific Ltd, Staffordshire, United Kingdom; www.barloworld-scientific.com). The 24 primer pairs used (S1 Table) to characterize the allelic variation of *CKX* genes in wheat were designed using DNAMAN software (v.6.0), based on the EST and mRNA sequences (refer to the note for S1 Table) retrieved from the NCBI (www.ncbi.nlm.nih.gov) [37].

The PCR profile was as follows: denaturation at 94°C for 5 min; followed by 40 cycles of denaturation at 95°C for 1 min; annealing at 50–60°C for 1 min 20 s; and extension at 72°C for 2 min, with a final extension for 8 min at 72°C. The annealing temperature varied according to the primer pair (S1 Table). Each 15-μl PCR reaction mixture contained 40 ng genomic DNA, 10 pmol each primer, 200 mM dNTP in 1 × PCR buffer, and 1 U *Taq* DNA polymerase (Shanghai Sangon Biological Engineering Technology & Services Co., Ltd., Shanghai, China; www.sangon.com). PCR products were separated on 7% PAGE containing 4 M urea.

### Sequencing of PCR Fragments and Statistical Analyses

PCR product fragments with the expected size were recovered from two independent samples per line. Target fragments were cloned into the pGEM-T vector, and sequenced from both strands by the Shanghai Sangon Biological Engineering Technology & Services Co. Ltd (http://www.sangon.com). Sequence alignments and characterizations were conducted using DNAMAN software. Analyses of chlorophyll content and grain weight data were conducted using SPSS software (v.13.0) (www.spss.com).

### Localization of *CKX* Gene on Chromosome

Chinese Spring nullisomic–tetrasomics were used to locate the *Tackx* gene. The PCR amplification and separation of products were conducted as described above.

### Quantitative Trait Loci Analysis for Grain Weight and Flag Leaf Chlorophyll Content

SSR markers were used to screen the two parents and two bulks, each containing five high-phenotypic-value-lines and five low-value-lines, respectively. To confirm the polymorphism, candidate polymorphic markers were analyzed further in a subset of 40 RIL, comprising 20 lines with high value in phenotype and 20 lines with low value in phenotype. The confirmed polymorphic markers were used to genotype the entire RIL. A linkage map was constructed using Map Manager QTXb20 (v.3.0). Recombination fractions were converted into centiMorgans (cM) using the Kosambi function [38]. Composite interval mapping (CIM) for quantitative trait loci (QTL) was analyzed using Windows Cartographer 2.5 software, according to the methods described by Zeng [39, 40]. An LOD score greater than 2.5 in at least two cropping seasons, calculated from 2,000 permutations at a probability of 0.01, indicated QTL existence.

### Correlation Analyses between *Tackx* Variation and Chlorophyll Content, and Grain Weight

When analyzing the correlation between *Tackx* alleles and chlorophyll content, and grain weight in the RILs, the Jing 411 allele was scored as “1” and the Hongmangchun 21 allele as “0”. The presence or absence of the *Tackx* genotype in each variety was scored as “1” or “0”, respectively. A Spearman’s correlation analysis and t-test were performed to test the significance of the association between *Tackx* variation and traits, as described in our previous studies [41, 42]. The effect of *Tackx* on phenotypic variation was estimated by *R* ^*2*^ using the general linear model (GLM) [41, 42]. Significance was evaluated using the model at the 0.05, 0.01, and 0.001 levels of probability. Data analyses were conducted using SPSS software (v.13.0).

## Results

### Grain Weight and Chlorophyll Content

The averaged TGW of Jing 411 over four cropping seasons was 46.2 g, while the averaged TGW of Hongmangchun 21 is 20.9 g. The two parents showed significant difference in grain yield, flag leaf chlorophyll content, and grain weight (Table 1). The natural population covering 102 wheat cultivars also showed great difference in flag leaf chlorophyll content and grain weight (Table 1).

### Correlations between Chlorophyll Content and Grain Weight

In the RIL population, the flag leaf chlorophyll content and grain weight showed high variations (Table 1). A correlation analysis was conducted between chlorophyll content at different stages after anthesis and grain weight, based on the mean value across four cropping seasons. The result indicated that flag leaf chlorophyll content had a significantly positive correlation with grain weight (*p* < 0.001) at 5 (0.523***), 10 (0.518***), and 15 (0.366***) DAA; however, no significant relationship between flag leaf chlorophyll content and grain weight was detected from 20 to 25 DAA (S2 Table). Therefore, when chlorophyll content was compared during early- and late-stage grain filling, early grain-filling stage chlorophyll content had a stronger effect on grain weight. Additionally, a significantly positive correlation occurred between chlorophyll contents of the two adjacent stages, e.g., C5 (5 DAA) and C10 (10 DAA), as shown in S2 Table, suggesting that grain weight can be improved via increase of chlorophyll content in flag leaf at early-stage of grain filling in wheat.

### Association of *Tackx* Genes with Chlorophyll Content and Grain Weight

**Fig 1:**
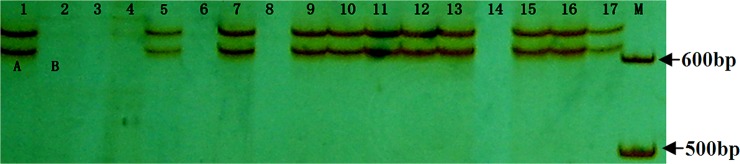
Allelic variation of *Tackx4* in the RILs population derived from Jing 411 × Hongmangchun 21. Lanes 1–17: 1, Jing 411; 2, Hongmangchun 21; 3, JH2; 4, JH17; 5, JH3; 6, JH11; 7, JH7; 8, JH15; 9, JH12; 10, JH13; 11, JH14; 12, JH20; 13, JH21; 14, JH19; 15, JH25; 16, JH26; 17, JH27. The genotype of Jing 411 (genotype-A) and Hongmangchun 21 (genotype-B) was marked “A” and “B” genotype, respectively. JH2, JH13, JH11, JH12, etc., represented individual names of the RILs.

Through the analysis of spearman rank correlation and t-test, *Tackx4*, amplified by T19-20 primer pair, was identified to have a significant association with chlorophyll content of flag leaf and grain weight among these *Tackx* genes (Table 2). In the RIL population, two patterns of *Tackx4* were detected and designated as genotype-A as in Jing 411 and genotype-B as in Hongmangchun 21, respectively (Fig 1). Genotype-A carried two fragments approximately 600bp in size, but genotype-B had no products amplified (Fig 1). Furthermore, the two fragments of *Tackx4* showed co-segregation, and no single fragment was found in the RIL. Spearman’s correlation analyses revealed that the fragment number variation of *Tackx4* was significantly correlated with both chlorophyll content of flag leaf from 5~15 DAA (*p*<0.01) and grain weight (*p*<0.001) (Table 2). A t-test analysis showed that the chlorophyll contents at 5~15 DAA and grain weight were significantly higher in the RIL individuals carrying genotype-A than those in genotype-B (Table 2). These results indicated that genotype-A is associated with higher chlorophyll content and grain weight.

**Table 2:** Statistical analysis of chlorophyll content and grain weight between two genotypes, and Spearman’s correlation analyses between allelic variation in *Tackx4* and related traits in RILs.

Trait	C5	C10	C15	C20	C25	TGW
*Tackx4* ^a^	0.347***	0.301***	0.288**	0.161	0.090	0.398***
t-test^b^	4.615***	5.137***	4.314**	2.018	1.561	6.383***

a: Significance at levels of 0.05, 0.01, and 0.001 is indicated with *, **, and ***, respectively.

b: *t*-test of averaged grain traits between two genotypes (genotype-A and genotype-B) of *Tackx4*.

### Chromosome Location of *Tackx4*

**Fig 2:**
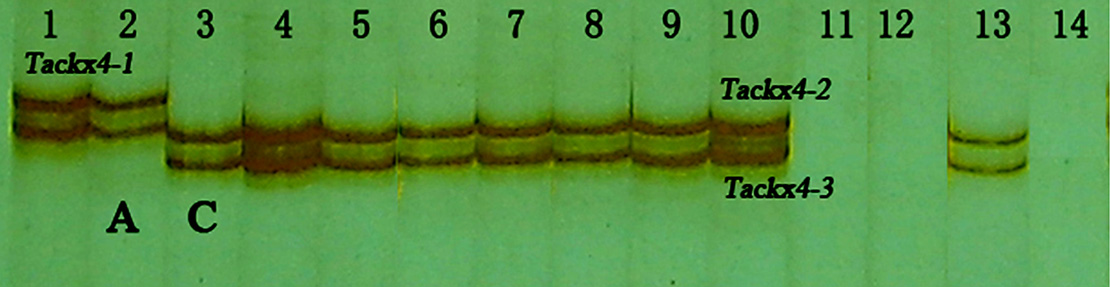
Amplified patterns of *Tackx4* from Chinese Spring nullisomic—tetrasomics using *T19-20* primer pair. Lanes 1–14: 1, Jing411; 2, Yumai 8679; 3, Yongchuanbaikemai (Chinese landrace); 4, Chinese spring; 5, Yongchuanbaimaizi (Chinese landrace); 6, Wanxianbaizi (Chinese landrace); 7, Heshangmai; 8, N3BT3A; 9, N3DT3A; 10, N3BT3D; 11, N3AT3D; 12, N3AT3B; 13,Yangmai 158; 14, Y6 (*Aegilops tauschii*, DD). The genotype-A (*Tackx4-1* and *Tackx4-2*) and genotype-C (*Tackx4-2* and *Tackx4-3*) are marked with A and C, respectively.

Previous studies have indicated that wheat *CKX* genes belong to a large gene family whose members are distributed on 3A, 3B, 3D, 7A, and 7B. In order to locate *Tackx4* before conducting a genetic linkage analysis in the RILs, Chinese Spring nullisomic–tetrasomics were used to locate *Tackx4* onto chromosome. Two fragments amplified with T19-20 primer pair, *Tackx4-2* and *Tackx4-3*, were detected in Chinese Spring (Fig 2), and this genotype was designated as genotype-C. *Tackx4* products were not amplified from N3AT3B, N3AT3D, and *Aegilops tauschii* (DD genome), but were amplified from the other nullisomic—tetrasomics (Fig 2), indicating that *Tackx4* is on chromosome 3A of common wheat. These results also revealed that at least three fragments could be amplified by the primer pair *T19-20* designed from *Tackx4* in common wheat.

### Linkage Analysis of *Tackx4* and Gene-Specific Marker Development

**Fig 3:**
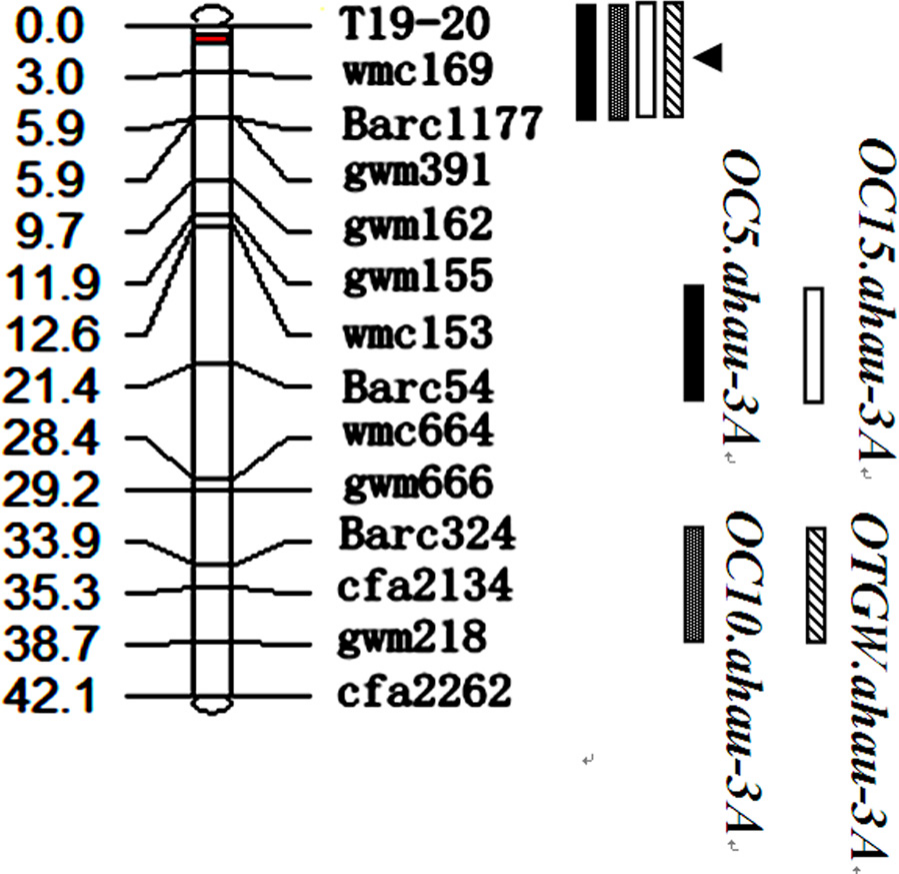
Linkage map of *Tackx4* on chromosome 3A of wheat. The four shaded boxes represented the four traits (C5, C10, C15 and TGW, respectively).

To analyze the genetic linkage of *Tackx4* on 3A and further evaluate its effect on phenotypic variations in chlorophyll content and grain weight, 49 SSR markers on chromosome 3A were used to screen the two parents (Jing 411 and Hongmangchun 21) and two bulks. Of the 49 markers, 13 SSR markers and *T19-20* showed polymorphisms and were located on the same linkage group, spanning a genetic distance of 42.1 cM (Fig 3).

QTL analysis identified a locus controlling both chlorophyll content of the flag leaf from 5~15 DAA and grain weight. This QTL was located at the interval between marker T19-20 and *wmc*169 on chromosome 3A, with a genetic distance of 3 cM (Fig 3). LOD values ranged from 4.5 to 8.3 across four cropping seasons. No QTL underlying chlorophyll content at 20 (C20) and 25 DAA (C25) was detected in this RIL population. QTL mapped at *Tackx4* locus was detected in all four cropping seasons, and explained 8.9%-22.3% of the phenotypic variation in chlorophyll content and grain weight (Table 3).

**Table 3:** Summary of QTL for grain weight across four cropping seasons in RILs derived from Jing 411 × Hongmangchun 21.

Trait^a^	QTL	Marker interval	Closest marker	LOD	*R* ^*2*^ (%)	Add. (%)	Environments observed/total
C5	*QC5*.*ahau-3A*	*T19-20~Xwmc169*	*T19-20*	7.9	20.1	-2.63	4/4
C10	*QC10*.*ahau-3A*	*T19-20~Xwmc169*	*T19-20*	6.8	13.2	-2.76	4/4
C15	*QC15*.*ahau-3A*	*T19-20~Xwmc169*	*T19-20*	4.5	8.9	1.21	4/4
TGW	*QTGW*.*ahau-3A*	*T19-20~Xwmc169*	*T19-20*	8.3	22.3	-0.48	4/4

a: The mean value from all seasons was used for analysis.

### Association Analysis between Variation of *Tackx4* and Chlorophyll Content of Flag Leaf and Grain Weight

**Fig 4:**
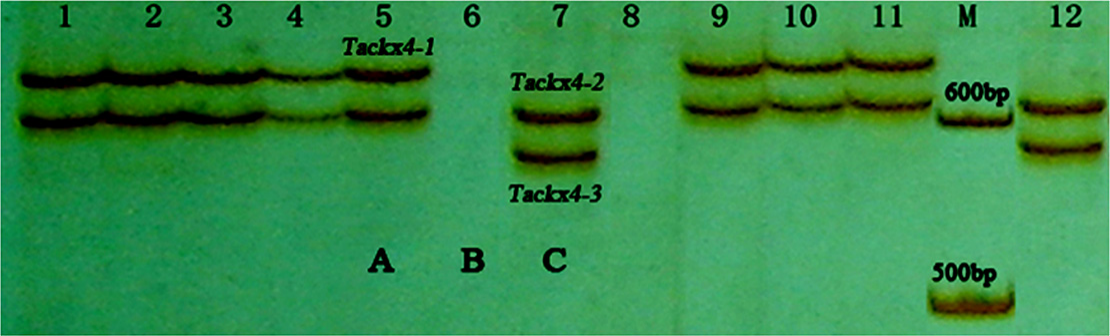
Amplification patterns of *Tackx4* gene for 12 of the 102 wheat varieties using *T19-20* primer pair. Lanes 1–12: 1, Jing411; 2, Yumai 8679; 3, Jimai 20; 4, Zhongmai 9; 5, Zheng 9023; 6, Hongmangchun 21; 7, Wanxianbaimaizi; 8, Wangshuibai; 9, Shan 225; 10, Shan 160; 11, Yumai 8679; 12, Xiaobingmai 33.

To further confirm the effects of *Tackx4* variations on the chlorophyll content after anthesis and grain weight, 102 different wheat varieties were genotyped and the relationship between their genotypes and grain weight were analyzed. Of the 102 varieties, 49 varieties carried genotype-A, 15 carried genotype-B, and 38 carried genotype-C (Fig 4).

Wide variations existed in chlorophyll content and grain weight among the 102 wheat varieties (Table 1). The mean values of these traits were significantly different (*p*<0.01) between genotype-A and genotype-B or genotype-C (Table 4). Association analysis between genotypes of *Tackx4* and chlorophyll content and grain weight found that C5, C10, C15, and grain weight were significantly positive correlated with genotype-A (*p*<0.01 or 0.001), but negatively correlated with genotype-B and genotype-C (Table 4). Compared with genotype-B and genotype-C, genotype-A could significantly help to improve chlorophyll content and grain weight. The phenotypic variations explained by each genotype ranged from 3.6% to 20.7% across the four cropping seasons.

**Table 4:** Spearman’s correlation between *Tackx4* genotypes and chlorophyll content, and grain weight

Trait^a^	*Tackx4* genotypes	No. of varieties	Mean of phenotype^b^	R/correlation ^c^	Effect ^d^ (%)
C5	A (*Tackx4-1/Tackx4-2*)	49	56.82±3.01C	0.412***	17.4
	B (*null*)	15	47.23±2.98B	-0.214*	5.1
	C (*Tackx4-2/Tackx4-3*)	38	43.64±3.77A	-0.335**	11.33
C10	A (*Tackx4-1/Tackx4-2*)	49	53.16±3.82C	0.397***	16.01
	B (*null*)	15	45.27±3.64B	-0.189*	3.60
	C (*Tackx4-2/Tackx4-3*)	38	40.56±2.77A	-0.287**	8.24
C15	A (*Tackx4-1/Tackx4-2*)	49	46.29±4.01B	0.312**	9.73
	B (*null*)	15	40.36±3.54A	-0.097	ns
	C (*Tackx4-2/Tackx4-3*)	38	38.43±3.12A	-0.261**	6.78
TGW	A (*Tackx4-1/Tackx4-2*)	49	41.07±3.24B	0.461***	20.7
	B (*null*)	15	33.83±2.79A	-0.198*	3.90
	C (*Tackx4-2/Tackx4-3*)	38	32.95±3.01A	-0.368**	12.4

a: Mean values were used for analysis.

b: Different letters in this column indicated significant differences (P<0.01; Fisher’s protected LSD) among different alleles

c: Significance at the levels of 0.05, 0.01 and 0.001 is indicated with *, **, and ***, respectively.

d: Effect of genotype of *Tackx4* on variance of phenotype; "ns", not significant.

### Sequence Analysis of Three Copies of *Tackx4*

**Fig 5:**
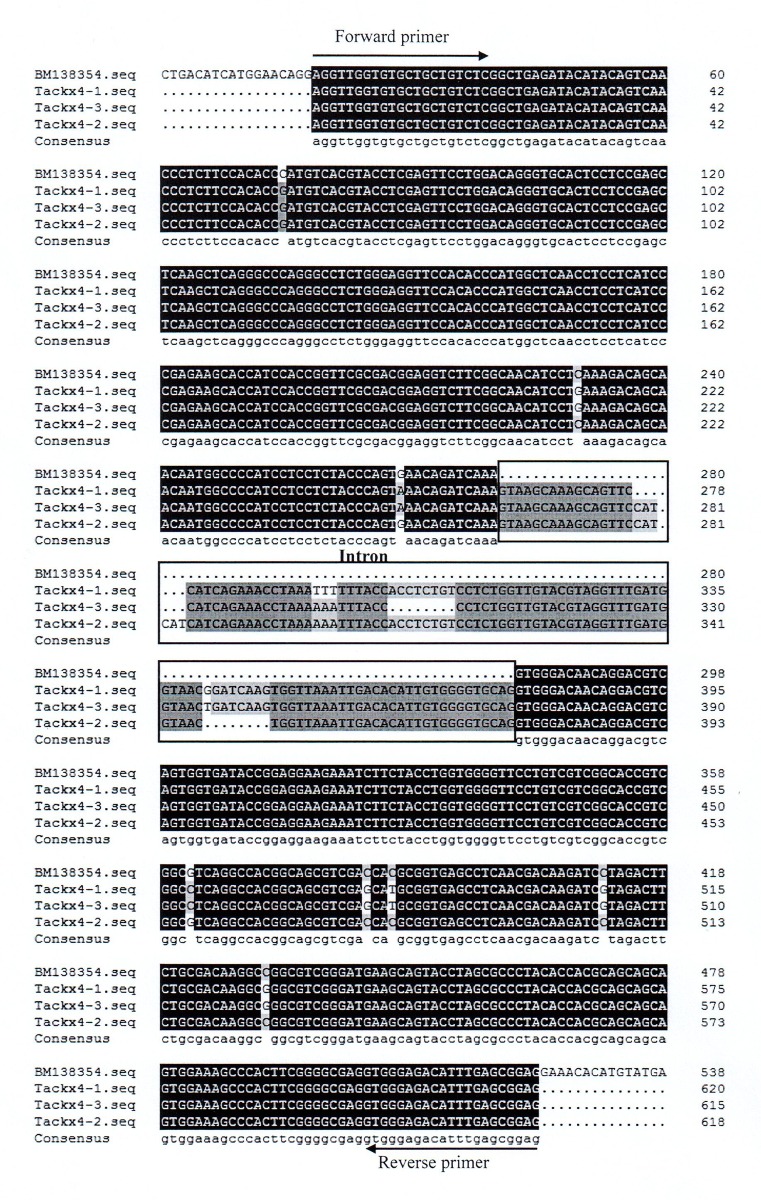
Sequence alignment of *Tackx4-1*, *Tackx4-2*, *Tackx4-3*, and *Tackx4b* (BM138354). Primer pair sequences and intron regions were marked. The primer pair was marked with arrows and the intron sequence was boxed.

**Fig 6:**
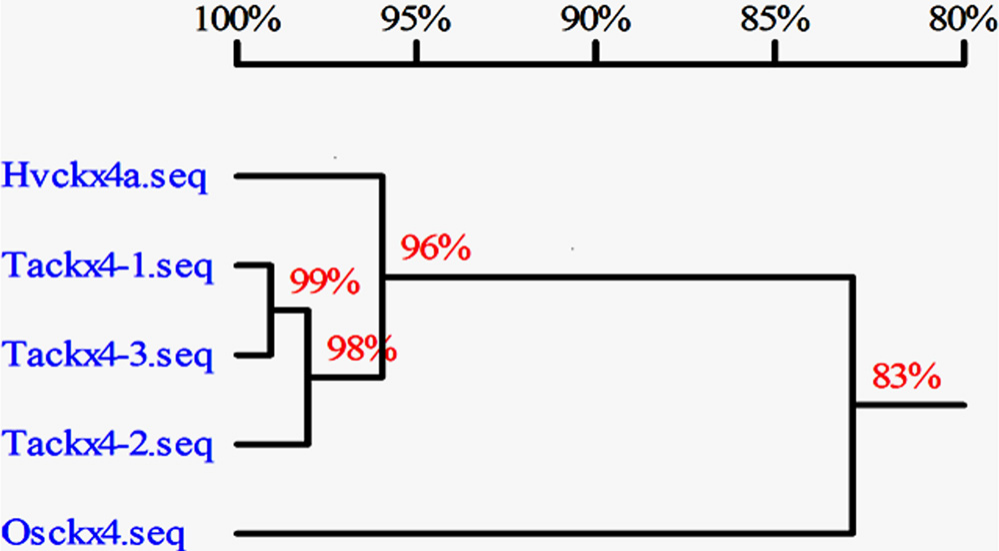
Homology analysis of *Tackx4* from wheat (*Tackx4-1*, *Tackx4-2* and *Tackx4-3*), *Hvckx4a* from barley, and *Osckx4* from rice. The percentages indicated the sequence identity among them.

Three fragments, *Tackx4-1*, *Tackx4-2*, and *Tackx4-3*, were sequenced and analyzed. These fragments showed high identity (>99%) with a wheat EST of *Tackx4* (BM138354), which was used to design the primers in this research. In addition, a high identity (>98%) among the sequences from these fragments themselves was also observed (Figs 5 and 6). The sequence analysis confirms that the *Tackx4* gene (BM138354) reported by Galuszka *et al*. [38] was one of the three fragments. Variations among sequences of three copies were detected in the intron at positions from 262bp to 378bp, which have the typical extron-intron boundary sequence “GT/AG”. Two 8-bp length InDels and an “AAA-TTT” variation were also found in this intron (Fig 5). Several SNPs in the exon region among the three sequences were observed.

*CKX* orthologous often exists in cereal plants. In this research, we obtained sequences of homologous genes of *CKX* from barley (*Hvckx4a*/BJ479455) and rice (*Osckx4*/XM_006645247) by BLAST searches in NCBI (www.ncbi.nlm.nih.gov), using the three sequences of *Tackx4* as the search query. Sequence analysis indicated that *Tackx4* had higher homology with barley *Hvckx4a* (>96%) than with rice *Osckx4* (>83%) (Fig 6). These results were generally consistent with previous report [37].

## Discussion

### Multiple Copies of *Tackx4* in Common Wheat

In previous studies, multiple copies of *CKX* genes have been identified on chromosome 3DS of wheat, including *Tackx2*.*1* and *Tackx2*.*2* [28], and *Tackx6* [29]. In the present study, at least three fragments of *Tackx4* with high sequence identity were identified on chromosome 3A using linkage and Chinese Spring nullisomic–tetrasomics analysis. The two fragments (*Tackx4-1* and *Tackx4-2*), showed co-segregation in the RIL population, and no single gene (*Tackx4-1* or *Tackx4-2*) was found. Additionally, these fragments always occurred in couples in the natural population. Therefore, our results suggested that it is copy number variation in *Tackx4*, but not allelic variation. The allelic variations in *CKX* gene are often observed in wheat (*Tackx2* and *Tackx6*) and rice (*Osckx2*) [26, 28, 29]; however, copy number variation of *CKX* is rarely reported.

Previous studies have shown that gene clusters, that is, multiple copies of genes with the same or similar functions, often occur in higher plants. The variations in copy numbers or the copy itself will affect gene cluster function [43–47]. In this study, copy number variations (CNV) in *Tackx4* could significantly influence wheat chlorophyll content and grain weight. The genotype-A generally corresponded to higher wheat chlorophyll content and grain yield, compared with genotype-B and genotype-C. As for genotype-B, no product was amplified around 600bp size, but it does not mean *Tackx4* was null because only partial length was analyzed in this study. Therefore, full length of *Tackx4* should be further studied. Some variations were also identified among the three copies of *Tackx4*, which is in consistent with previous report [45–47].

### Sequence Analyses of Three *Tackx4* Copies

Mutations in the coding regions of genes can affect gene function as a result of changes in its amino acid sequence. However, mutations in the 5' UTR [48, 49], the 3' UTR [50–52], or introns [29, 53–55] can also affect gene function as a result of changes in mRNA structure, stability, and accumulation during translation. Zhang et al. [29] showed that an 18-bp InDel in the second intron of *Tackx6* significantly affected its transcript level at 8 days after pollination. In the present study, two 8-bp indels and an "AAA" mutation were also detected in the intron of *Tackx4*. Hence it should be further investigated whether variations of these copies themselves have effect on their transcription and translation. In addition, whether the CNV of *Tackx4* affects the chlorophyll content of the flag leaf and grain weight through directly regulating the level of endogenous cytokinin is also worthy of exploration in the future. Several SNPs in the coding region of *Tackx4* may affect its function.

As previous reports [29, 37], *CKXs* orthologous widely exist in cereals, e.g., wheat, barley, rice, and maize. *Tackx4* on 3A of wheat had high identity in sequence with *Osckx4* on chromosome 1 of rice, which is in consistence with genome synteny between them. However, the function of *Osckx4* is still unknown.

### *Tackx4* Locus for Chlorophyll Content and Grain Weight

In previous studies, several QTLs for yield and yield related traits have been detected on wheat chromosome 3A. These QTLs were able to explain 4.1~14.27% of phenotypic variations in different environments [56–60]. In this study, *Tackx4* locus was mapped on chromosome 3A, and closely linked to *wmc*169, which located at different linkage intervals from those of the QTLs reported previously. As for chlorophyll content and photosynthesis, many QTLs have been found on chromosomes 1A, 1D, 2A, 2D, 3B, 4A, 5A, 5B, 5D, 6A, 6D, 7A, and 7D [61–65]. In our study, the QTL co-segregating with *Tackx4* on 3A was different from the previous reports and could be considered as a novel locus for chlorophyll content of the flag leaf and grain weight. The novel locus *Tackx4* can be used as a molecular marker for genetic improvement of grain weight in wheat breeding program.

In high plant, photosynthetic efficiency of leaf is directly influenced by the content and stability of chlorophyll [4–5]. Therefore, increased chlorophyll content in leaves can improve grain filling and yield [6–10]. In this study, the chlorophyll contents of flag leaf at 5~15 DAA had a close relationship with grain weight, which is generally consistent with previous studies. Here, *Tackx4* was confirmed to be significantly associated with these traits simultaneously. According to these analyses, *Tackx4* could be presumed to involve in controlling grain weight through regulating leaf chlorophyll and photosynthesis, which is stabilized by CTK. Therefore, identification of *Tackx4* function will deepen our understanding molecular mechanisms of chlorophyll content and grain weight. Furthermore, the locus linked to *Tackx4* showed good stability and reliability in varied environments and genetic backgrounds. These attributes will be useful for improving the accuracy and effectiveness of marker assisted selection (MAS) for chlorophyll level and grain weight in wheat breeding.

## Supporting Information

S1 Table Primer pairs used in this study. (DOC)

S2 Table Correlations between chlorophyll content of flag leaf and thousand-grain-weight of RILs across different cropping seasons. (DOC)

## References

[1] Evans LT (1993) Crop evolution, adaption and yield New York: Cambridge.

[2] Austin RB, Edrich JA, Ford MA, Blackwell RD (1977) The fate of the dry matter, carbohydrates and C lost from the leaves and stems of wheat during grain filling. Ann Bot 41: 1309–1321.

[3] Bidinger F, Musgrave RB, Fisher RA (1977) Contribution of stored pre-anthesis assimilate to grain yield in wheat and barley. Nature 270: 431–433.

[4] Araus JI, Bort J, CecCadelli S and Grando S (1997) Relationship between leaf structure and carbon isotope discrimination in field grown barley. Plant Physiol Biochem 35: 533–541.

[5] Thomas JA, Jeffrey AC, Atsuko K and David MK (2005) Regulating the proton budget of higher plant photosynthesis. Proc Natl Acad Sci 102: 9709–9713. 15972806 10.1073/pnas.0503952102 PMC1172270

[6] Wang FH, Wang GX, Li XY, Huang JL and Zheng JK (2008) Heredity, physiology and mapping of a chlorophyll content gene of rice (*Oryza sativa* L.). J Plant Physiol 165: 324–330. 17306416 10.1016/j.jplph.2006.11.006

[7] Fisher RA, Rees D, Sayre KD, Lu ZM, Condon AG, Larque SA (1998) Wheat yield progress associated with higher stomatal conductance and photosynthetic rate, and cooler canopies. Crop Sci 38: 1467–1475.

[8] Ramesh K, Chandrasekaran1 B, Balasubramanian TN, Bangarusamy U, Sivasamy R, Sankaran N (2002) Chlorophyll dynamics in rice (*Oryza sativa*) before and after flowering based on SPAD (Chlorophyll) meter monitoring and its relation with grain yield. J Agron Crop Sci 188 (2): 102–105.

[9] Zhao H, Dai TB, Jing Q, Jiang D, Cao WX (2007) Leaf senescence and grain filling affected by post-anthesis high temperatures in two different wheat cultivars. Plant Growth Regul 51: 149–158.

[10] Shao GQ, Li ZJ, Ning TY, Zheng YH (2013) Responses of photosynthesis, chlorophyll fluorescence, and grain yield of maize to controlled-release urea and irrigation after anthesis. J Plant Nutrition and Soil Sci 176 (4): 595–602.

[11] Fletcher RA and McCullagh D (1971) Cytokinin-Induced chlorophyll formation in cucumber cotyledons. Planta (Berl.) 101: 88–90. 24488296 10.1007/BF00387693

[12] Catsky J, Pospisilova J, Machackova I, Wilhelmova N and Sestak Z (1993) Photosynthesis and water relation in transgenic tobacco plants with T-DNA carrying gene 4 for cytokinin synthesis. Biol Plantarum 35: 393–399.

[13] Clarke SF, Jameson PE, Downs C (1994) The influence of 6-benzylaminopurine on post-harvest senescence of floral tissues of *broccoli* (*Brassica oleraea* var Italia). Plant Growth Regul 14: 21–27.

[14] Wingler A, von Schaewen A, Leegood RC, Lea PJ, Quick WP (1998) Regulation of leaf senescence by cytokinin, sugars and light. Plant Physiol 116: 329–335.

[15] Khamlichi CR, Huntley R, Jacqmard A, James AH (1999) Cytokinin activation of arabidopsis cell division through a D-type cyclin. Science 283: 1541–1544. 10066178 10.1126/science.283.5407.1541

[16] Gupta NK, Gupta S, Kumar A (2000) Exogenous cytokinin application increases cell membrane and chlorophyll stability in wheat (*Triticum aestivum* L.). Cereal Res Commu 28(3): 287–291.

[17] Lee EG, Seo JS, Cha MH, Suh MC, and Sim WS (2002) Cytokinin stimulates expression of the chloroplast ATP synthase IV subunit gene (*atpl*). J Plant Biol 45(2): 71–76.

[18] Ookawa T, Naruoka Y, Sayama A and Hirasawa T (2004) Cytokinin effects on ribulose-1, 5-bisphosphate carboxylase/oxygenase and nitrogen partitioning in rice during ripening. Crop Sci 44: 2107–2115.

[19] An JS, Zhang M, Lu QR, Zhang ZG (2006) Effect of a prestorage treatment with 6-benzylaminopurine and modified atmosphere packaging storage on the respiration and quality of green asparagus spears. J Food Eng 77: 951–957.

[20] Shao RX, Yang QH, Xin LF, Shangguan ZP (2012) Effects of exogenous nitric oxide and cytokinin on the growth and photosynthesis of wheat seedlings under water deficit. J Food Agri & Environ 10: 1451–1456.

[21] Ding XT, Jiang YP, Wang H, Jin HJ, Zhang HM, Chen CH, et al (2013) Effects of cytokinin on photosynthetic gas exchange, chlorophyll fluorescence parameters, antioxidative system and carbohydrate accumulation in cucumber (*Cucumis sativus* L.) under low light. Acta Physiol Plant 35: 1427–1438.

[22] Swartzberg D, Kirshner B, Rav-David D, Elad Y & Granot D (2008) *Botrytis cinerea* induces senescence and is inhibited by autoregulated expression of the *IPT* gene. Eur J Plant Pathol 120: 289–297.

[23] Ma QH and Liu YC (2009) Expression of isopentenyl transferase gene (*ipt*) in leaf and stem delayed leaf senescence without affecting root growth. Plant Cell Rep 28: 1759–1765. 10.1007/s00299-009-0776-1 19820948

[24] Todorova D, Vaseva-Gemisheva I, Petrov P, Stoynova-Bakalova E, Alexieva V, Karanov E, et al (2006) Cytokinin oxidase/dehydrogenase (CKX) activity in wild and ethylene-insensitive mutant *eti5* type of Arabidopsis thaliana (L.) Heynh plants and the effect of cytokinin N1-(2-chloro-4-pyridyl)-N2-phenylurea on enzymatic activity and leaf morphology. Acta Physi Plantarum 28: 613–617.

[25] Schlütera T, Leideb J, Conradc K (2011) Light promotes an increase of cytokinin oxidase/dehydrogenase activity during senescence of barley leaf segments. J Plant Physio 168: 694–698. 10.1016/j.jplph.2010.10.004 21106275

[26] Ashikari M, Sakakibara H, Lin SY, Yamamoto T, Takashi T, Nishimura A, et al (2005) Cytokinin oxidase regulates rice grain production. Science 309: 741–745. 15976269 10.1126/science.1113373

[27] Zalewski W, Galuszka P, Gasparis S, Orczyk W and Nadolska-Orczyk A (2010) Silencing of the HvCKX1 gene decreases the cytokinin oxidase/dehydrogenase level in barley and leads to higher plant productivity. J Exp Bot 61: 1839–1851. 10.1093/jxb/erq052 20335409

[28] Zhang JP, Liu WH, Yang XM, Gao AN, Li XQ, Wu XY, et al (2011) Isolation and characterization of two putative cytokinin oxidase genes related to grain number per spike phenotype in wheat. Mol Biol Rep 38: 2337–2347. 10.1007/s11033-010-0367-9 21104150

[29] Zhang L, Zhao YL, Gao LF, Zhao GY, Zhou RH, Zhang BS, et al (2012) *TaCKX6-D1*, the ortholog of rice *OsCKX2*, is associated with grain weight in hexaploid wheat. New Phytol 195: 574–584. 10.1111/j.1469-8137.2012.04194.x 22670578

[30] Feng DS, Wang HG, Zhang XS, Kong LR, Tian JC, Li XF (2008) Using an inverse PCR method to clone the wheat cytokinin oxidase/dehydrogenase gene *TaCKX1* . Plant Mol Biol Rep 26: 143–155.

[31] Zhang L, Zhang BS, Zhou RH, Gao LF, Zhao GY, Song YX, et al (2007) Cloning and genetic mapping of cytokinin oxidase/dehydrogenase gene (*TaCKX2*) in Wheat. Acta Agron Sin 33: 1419–1425.

[32] Ma X, Feng DS, Wang HG, Li XF, Kong LR (2011) Cloning and expression analysis of wheat cytokinin oxidase/dehydrogenase gene *TaCKX3* . Plant Mol Biol Rep 29: 98–105.

[33] Zhang L, Zhang B, Zhou R, Kong X, Gao L, Jia JZ (2008) Isolation and chromosomal localization of cytokinin oxidase/dehydrogenase gene (*TaCKX5*) in wheat. Sci Agri Sin 41: 636–642.

[34] Wu FB, Wu LH, Xu FH (1998) Chlorophyll meter to predict nitrogen side dress requirements for short-season cotton (*Gossypium hirsutum* L.). Field Crops Res 56: 309–314.

[35] Rosyara UR, Subedi S, Duveiller E, Sharma RC (2010) The effect of spot blotch and heat stress on variation of canopy temperature depression, chlorophyll fluorescence and chlorophyll content of hexaploid wheat genotypes. Euphytica 174: 377–390.

[36] Kang HW, Cho YG, Yoon UH (1998) A rapid DNA extraction method for RFLP and PCR analysis from a single dry seed. Plant Mol Biol Rep 16: 1–9.

[37] Galuszka P, Frébortová J, Werner T, Yamada M, Strnad M, Schmülling T, et al (2004) Cytokinin oxidase/dehydrogenase genes in barley and wheat Cloning and heterologous expression. Eur J Biochem 271: 3990–4002. 15479228 10.1111/j.1432-1033.2004.04334.x

[38] Manly KF, Cudmore RH Jr, Meer JM (2001) Map Manager QTX, cross-platform software for genetic mapping. Mamm Genome 12: 930–932. 11707780 10.1007/s00335-001-1016-3

[39] Zeng ZB (1993) Theoretical basis for separation of multiple linked gene effects in mapping quantitative trait loci. Proc Natl Acad Sci 90 (23): 10972–10976. 8248199 10.1073/pnas.90.23.10972 PMC47903

[40] Zeng ZB (1994) Precision mapping of quantitative trait loci. Genetics 136 (4): 1457–1468. 8013918 10.1093/genetics/136.4.1457 PMC1205924

[41] Zhang HP, Chang C, You GX, Zhang XY, Yan CS, Xiao SH, et al (2010) Identification of molecular markers associated with seed dormancy in mini core collections of Chinese wheat and landraces. Acta Agron Sin 36: 1649–1656.

[42] Chang C, Zhang HP, Zhao QX, Feng JM, Si HQ, Lu J, et al (2011) Rich allelic variations of *Viviparous-1A* and their associations with seed dormancy/pre-harvest sprouting of common wheat. Euphytica 179: 343–353.

[43] Simons G, Groenendijk J, Wijbrandi J, Reijans M, Groenen J, Diergaarde P, et al (1998) Dissection of the fusarium I2 gene cluster in tomato reveals six homologs and one active gene copy. The Plant Cell 10: 1055–1068. 9634592 10.1105/tpc.10.6.1055 PMC144031

[44] Han YB, Gasic K and Korban SS (2007) Multiple-copy cluster-type organization and evolution of genes encoding *O*-Methyltransferases in the apple. Genetics 176: 2625–2635. 17717198 10.1534/genetics.107.073650 PMC1950660

[45] Huynh BL, Mather DE, Schreiber AW, Toubia J, Baumann U, Shoaei Z, et al (2012) Clusters of genes encoding fructan biosynthesizing enzymes in wheat and barley. Plant Mol Biol 80: 299–314. 10.1007/s11103-012-9949-3 22864927

[46] Hanikenne M, Kroymann J, Trampczynska A, Bernal M, Motte P, Clemens S, et al (2013) Hard selective sweep and ectopic gene conversion in a gene cluster affording environmental adaptation. PLOS Genetics 9: e1003037. 10.1371/journal.pgen.1003707 PMC3749932 23990800

[47] Zmienko A, Samelak A, Kozłowski P, Figlerowicz M (2014) Copy number polymorphism in plant genomes. Theor Appl Genet 127: 1–18. 10.1007/s00122-013-2177-7 23989647 PMC4544587

[48] Paradkar MR, Marcotte WR (2001) Changes in the wheat Em 5 ' UTR affect reporter gene expression in vivo and in vitro. J Plant Physiol 158: 929–934.

[49] Kamo K, Kim AY, Park SH, Joung YH (2012) The 5 ' UTR-intron of the Gladiolus polyubiquitin promoter GUBQ1 enhances translation efficiency in Gladiolus and Arabidopsis. BMC Plant Bio 12: 79. 22672685 10.1186/1471-2229-12-79 PMC3406973

[50] Monde RA, Greene JC, Stern DB (2000) The sequence and secondary structure of the 3 '-UTR affect 3 '-end maturation, RNA accumulation, and translation in tobacco chloroplasts. Plant Mol Bio 44 (4): 529–542. 11197327 10.1023/a:1026540310934

[51] Gandikota M, Birkenbihl RP, Hohmann S, Cardon GH, Saedler H, Huijser P (2007) The miRNA156/157 recognition element in the 3' UTR of the Arabidopsis SBP box gene SPL3 prevents early flowering by translational inhibition in seedlings. Plant J 49 (4): 683–693. 17217458 10.1111/j.1365-313X.2006.02983.x

[52] Vignesh M, Nepolean T, Hossain F, Singh AK, Gupta HS (2013) Sequence variation in 3'UTR region of crtRB1 gene and its effect on beta-carotene accumulation in maize kernel. J Plant Biochen and Biotech 22 (4): 401–408.

[53] Chang C, Zhang HP, Xu J, You MS, Li BY, Liu GT (2007) Variation in two PPO genes associated with polyphenol oxidase activity in seeds of common wheat. Euphytica 154:181–193.

[54] Chang C, Zhang HP, Feng JM, Yin B, Si HQ, Ma CX (2010) Identifying alleles of *Viviparous-1B* associated with pre-harvest sprouting in micro-core collections of Chinese wheat germplasm. Mol Breed 25:481–490.

[55] De La Torre CM, Finer JJ (2015) The intron and 5 ' distal region of the soybean Gmubi promoter contribute to very high levels of gene expression in transiently and stably transformed tissues. Plant Cell Rep 34 (1): 111–120. 10.1007/s00299-014-1691-7 25292438

[56] Huang XQ, Kempf H, Ganal MW, Rӧder MS (2004) Advanced backcross QTL analysis in progenies derived from a cross between a German elite winter wheat variety and a synthetic wheat (*Triticum aestivum* L.). Theor Appl Genet 109:933–943. 15243706 10.1007/s00122-004-1708-7

[57] Groos C, Robert N, Bervas E, Charmet G (2003) Genetic analysis of grain protein-content, grain yield and thousand-kernel weight in bread wheat. Theor Appl Genet 106: 1032–1040. 12671751 10.1007/s00122-002-1111-1

[58] Kumar N, Kulwal PL, Balyan HS, Gupta PK (2007) QTL mapping for yield contributing traits in two mapping populations of bread wheat. Mol Breed 19:163–177.

[59] Cuthbert JL, Somers DJ, Brule´-Babel AL, Brown PD, Crow GH (2008) Molecular mapping of quantitative trait loci for yield and yield components in spring wheat (*Triticum aestivum* L.). Theor Appl Genet 117: 595–608. 10.1007/s00122-008-0804-5 18516583

[60] Wang RX, Hai L, Zhang XY, You GX, Yan CS, Xiao XH (2009) QTL mapping for grain filling rate and yield-related traits in RILs of the Chinese winter wheat population Heshangmai ×Yu8679. Theor Appl Genet 118: 313–325. 10.1007/s00122-008-0901-5 18853131

[61] Yang DL, Jing RL, Chang XP, Li Wei (2007) Quantitative trait loci mapping for chlorophyll fluorescence and associated traits in wheat (*Triticum aestivum* L). J Integr Plant Biol 49 (5): 646–654.

[62] Zhang K, Zhang Y, Chen G, Tian J (2009) Genetic analysis of grain yield and leaf chlorophyll content in common wheat. Cereal Res Commu 37 (4): 499–511.

[63] Czyczyło-Mysza I, Tyrka M, Marcinska I, Skrzypek E, Karbarz M, Dziurka M, et al (2013) Quantitative trait loci for leaf chlorophyll fluorescence parameters, chlorophyll and carotenoid contents in relation to biomass and yield in bread wheat and their chromosome deletion bin assignments. Mol Breed 32: 189–210. 23794940 10.1007/s11032-013-9862-8 PMC3684715

[64] Czyczyło-Mysza I, Marcinska I, Skrzypek E, Chrupek M, Grzesiak S, Hura T, et al (2011) Mapping QTLs for yield components and chlorophyll a fluorescence parameters in wheat under three levels of water availability. Plant Genetic Res 9(2): 291–295.

[65] Sukumaran S, Dreisigacker S, Lopes M, Chavez P, Reynolds MP (2015) Genome‑wide association study for grain yield and related traits in an elite spring wheat population grown in temperate irrigated environments. Theor Appl Genet 128: 353–363. 10.1007/s00122-014-2435-3 25490985

