# Supplementary material for: Copy Number Variation of Cytokinin Oxidase Gene Tackx4 Associated with Grain Weight and Chlorophyll Content of Flag Leaf in Common Wheat
Source: PLoS One. 2015 Dec 29;10(12):e0145970. doi: 10.1371/journal.pone.0145970 (PMC4699907; doi:10.1371/journal.pone.0145970)
Supplement: S1 Table — (DOC) [file pone.0145970.s001.doc]

S1 Table. Primer pairs used in this study

| Primer name | *CKX* gene^a^ | Forward sequence 5’-3’ | Reverse sequence 5’-3’ | T^b^/℃ |
| --- | --- | --- | --- | --- |
| T1-2 | *Tackx1* | acgggtacatgctggagattaaag | aggtggcgctggacaagatc | 55 |
| T3-4 | *Tackx1* | gcagcctcttggggtcgtact | actcgggcgggttcttcac | 56 |
| T5-6 | *Tackx2a* | aggtgacatctttggttttctttg | cggagggcgaggtgttctac | 56 |
| T7-8 | *Tackx2a* | ggcctccgtgcggtagtgc | cacccgtggctcaacctcttc | 58 |
| T9-10 | *Tackx2b* | gctgctaatgcgcgatcttgc | atgcggcggctcaagtacgt | 55 |
| T11-12 | *Tackx2b01* | ccctttccctacgacaacatcca | cacccgtggctcaacctcttc | 56 |
| T13-14 | *Tackx3* | cacggcttgatgcatgcttcc | ccggagggcgaggtgttcta | 57 |
| T15-16 | *Tackx3* | ccctataatcatcgtcatcgtcct | ccggagggcgaggtgttcta | 56 |
| T17-18 | *Tackx4* | ccagttcggcatcatcaccag | tgtggaagagggttgactgtatgt | 55 |
| T19-20 | *Tackx4* | aggttggtgtgctgctgtctc | ctccgctcaaatgtctcccac | 55 |
| T21-22 | *Tackx4* | ggcgaggtgggagacatttga | tggtgggcttgtgtcgttactc | 55 |
| T23-24 | *Tackx5a* | cggtgaagttggagtagagcg | cggggtggtggtggacat | 55 |
| T25-26 | *Tackx5b* | tcggggccaggcagtacc | tgcgcgccacatacatgacac | 56 |
| T27-28 | *Tackx5b01* | cgcgacgcattcctctgtacat | ccccatgaacaagcacaagtg | 55 |
| T29-30 | *Tackx6a01* | gcctcctcgcagaatcgtaaga | ggttcgcgttcgtgcaggac | 56 |
| T31-32 | *Tackx6a02* | ccgggaccatgcaagcaa | acaggtgggacggcaaca | 54 |
| T33-34 | *Tackx6b* | cgggcagacacggggaac | gcaaagcgcgagaaatgacag | 55 |
| T35-36 | *Tackx6b01* | gtgcggacgcttgccctc | agcgcgtgcgagagagatga | 56 |
| T37-38 | *Tackx7a* | gggcgccagagaggtttatatg | ccggggtccaaggttcagg | 56 |
| T39-40 | *Tackx7b* | gcctccaagaatcactcactcac | ggcccgtgcttgaatgtctg | 55 |
| T41-42 | *Tackx7b* | gcggtgttgaagccccagtc | gggcgagcatgtcacggtct | 56 |
| T43-44 | *Tackx8* | ggggctcatcctcatctatcc | agggcaacatacagatcgaacag | 55 |
| T45-46 | *Tackx6b* | gcgcgagaaatgacagtgatag | cgggcagacacggggaac | 56 |
| T47-48 | *Tackx6b01* | cgatcgttcacccaagctaag | gggcagacaccggggaac | 56 |

a: Names of *Tackx* ESTs are according to Galuszka *et al.* [37], and their accessions no. in GenBank are AF362471, AL825717, AL822297 for *Tackx1*; BG905097 for *Tacx2a*; CD932650 for *Tackx2b*; BQ801874 for *Tackx2b01*; BE404516 for *Tackx3*; BM138354, BJ306089 for *Tackx4*; BM137409 for *Tackx5a*; BQ161648 for *Tackx5b*; CB877904 for *Tackx5b01*; CA705202, BQ903062 for *Tackx6a01*; BQ235927 for *Tackx6a02*; BQ238832 for *Tackx6b*; CA704486 for *Tackx6b01*; CA603337 for *Tackx7a*; BJ316444 for *Tackx7b*; BJ322935 for *Tackx8*, respectively.

b: Annealing temperature of primer pair.
