# Supplementary material for: Copy Number Variation of Cytokinin Oxidase Gene Tackx4 Associated with Grain Weight and Chlorophyll Content of Flag Leaf in Common Wheat
Source: PLoS One. 2015 Dec 29;10(12):e0145970. doi: 10.1371/journal.pone.0145970 (PMC4699907; doi:10.1371/journal.pone.0145970)
Supplement: S2 Table — (DOC) [file pone.0145970.s002.doc]

S2 Table. Correlations between chlorophyll content of flag leaf and thousand-grain-weight of RILs based on mean value across different cropping seasons

| Trait | C10 | C15 | C20 | C25 | TGW |
| --- | --- | --- | --- | --- | --- |
| C5 | 0.902*** | 0.572*** | 0.472*** | 0.016 | 0.523*** |
| C10 |  | 0.677*** | 0.533*** | 0.094 | 0.518*** |
| C15 |  |  | 0.641*** | 0.309** | 0.366*** |
| C20 |  |  |  | 0.588*** | 0.147 |
| C25 |  |  |  |  | 0.055 |

Significant correlations at probability levels of 0.05, 0.01 and 0.001 are marked with *, **, and ***, respectively; “ns”, not significant.
